# Targeted transcriptomic analysis of pancreatic adenocarcinoma in EUS-FNA samples by NanoString technology

**DOI:** 10.3389/fmolb.2023.1161893

**Published:** 2023-05-17

**Authors:** L. Pedrosa, I. K. Araujo, M. Cuatrecasas, G. Soy, S. López, J. Maurel, C. Sánchez-Montes, C. Montironi, T. Saurí, O. Sendino, F. M. Pérez, F. Ausania, G. Fernández-Esparrach, F. M. Espósito, E. C. Vaquero, A. Ginès

**Affiliations:** ^1^ Institut d’Investigacions Biomèdiques August Pi i Sunyer (IDIBAPS), Barcelona, Spain; ^2^ Endoscopy Unit, Gastroenterology Department, ICMDM, Hospital Clínic, Barcelona, Spain; ^3^ Pathology Department, Centre of Biomedical Diagnosis (CDB), Hospital Clínic, Barcelona, Spain; ^4^ Facultat de Medicina i Ciències de la Salut, University of Barcelona (UB), Barcelona, Spain; ^5^ Medical Oncology Department, Translational Genomics and Targeted Therapies in Solid Tumors, ICMHO, Hospital Clínic, Barcelona, Spain; ^6^ Centro de Investigación Biomédica en Red en Enfermedades Hepáticas y Digestivas (CIBEREHD), Barcelona, Spain; ^7^ Molecular Biology Core, CDB, Hospital Clinic, Barcelona, Spain; ^8^ Department of General and Digestive Surgery, ICMDM, Hospital Clínic, Barcelona, Spain; ^9^ Gastroenterology Department, ICMDM, Hospital Clínic, Barcelona, Spain

**Keywords:** pancreatic adenocarcinoma, EUS-FNA, NanoString technology, tumour stroma, transcriptomic analysis

## Abstract

**Background:** Integration of transcriptomic testing into EUS-FNA samples is a growing need for precision oncology in pancreatic ductal adenocarcinoma (PDAC). The NanoString platform is suitable for transcriptome profiling in low yield RNA samples.

**Methods:** Inclusion of patients that underwent EUS-FNA cytological diagnosis of pancreatic ductal adenocarcinoma using 19G and/or 22G needles and subsequent surgical resection. Formalin-fixed, paraffin-embedded (FFPE) cytological and surgical samples underwent RNA extraction and transcriptomic analysis using a custom 52-gene NanoString panel of stromal PDAC features. Cell type abundance was quantified in FFPE specimens and correlated.

**Results:** 18 PDAC patients were included. Mean EUS-FNA passes was 2 + 0.7. All FFPE passed the RNA quality control for genomic analysis. Hierarchical clustering on the global gene expression data showed that genes were differentially expressed between EUS and surgical samples. A more enriched cancer-associated fibroblasts and epithelial-mesenchymal transition transcriptomic profile was observed across surgical specimens whereas immunological biomarkers were more represented in EUS-FNA samples. Cytological examination confirmed a scanty representation of CAF and more immunological cell abundance in cytological samples in comparison to surgical specimens.

**Conclusion:** Targeted transcriptomic NanoString profiling of PDAC samples obtained by EUS-FNA is a feasible approach for pre-surgical molecular analysis although stromal CAF/EMT mRNA biomarkers are underrepresented.

## 1 Introduction

Despite major oncologic therapeutic avenues in the last decades, pancreatic ductal adenocarcinoma (PDAC) still carries a dismal prognosis, with a 5-year survival after diagnosis under 10% (Bray et al., 2018). Moreover, it is expected to become the second cause of death from cancer in the United States by 2030 (Rahib et al., 2021). The aggressive tumor nature, late diagnosis and limited therapeutic options are the main reasons for the poor outcome of the disease.

With the advent of the genomic era and precision medicine, genome-sequencing studies have provided a new perspective for the diagnosis, stratification, and treatment of PDAC (Casolino et al., 2021; Zheng-Lin and O’Reilly, 2021). Profiling gene signatures of PDAC has also become an attractive strategy to predict response to chemotherapy (Collisson et al., 2011; Moffitt et al., 2015; Bailey et al., 2016; Puleo et al., 2018) and to identify actionable molecular targets for precision medicine pipelines (Aung et al., 2018; Dreyer et al., 2020). Noteworthy, most of these results come from surgically resected specimens. This circumstance excludes patients with locally advanced or metastatic disease, which represent up to 80% at the time of diagnosis and might introduce a bias in the available data. On the other hand, there is increasing evidence that tumor stroma plays a critical role in PDAC development, progression, and therapy resistance (Neesse et al., 2019; Masugi, 2022). Indeed, different stromal components have emerged as prognostic biomarkers (Pu et al., 2019; Robin et al., 2020) and actionable targets (Hosein et al., 2020) in PDAC.

EUS-guided fine-needle aspiration (EUS-FNA) is the technique of choice for safely sampling the pancreas, with a pooled sensitivity and specificity for the diagnosis of PDAC of 85% and 98% respectively (Hewitt et al., 2012). Beyond cytopathological evaluation, several studies have shown excellent performance of EUS-FNA samples from PDAC for DNA genomic analysis (Larghi et al., 2020; Habib et al., 2021; Park et al., 2021). RNA analysis of EUS-acquired pancreatic samples is less extended than DNA based approaches, mainly because of the low yield and quality of RNA due to its easy degradation by pancreatic RNAases. Nevertheless, RNA profiling has been shown to be suitable in EUS-derived PDAC samples by using different approaches, such as real time quantitative polymerase chain reaction (RT-qPCR) (Archibugi et al., 2020), RNA Sequencing (RNA-Seq) (Rodriguez et al., 2016; Lundy et al., 2021), and digital mRNA analysis based on NanoString technology (Gleeson et al., 2020; Lundy et al., 2021; Rasmussen et al., 2021).

The NanoString nCounter analysis platform is an attractive choice to integrate transcriptome profiling on EUS-FNA samples (Tsang et al., 2017). This technology uses a digital fluorescent barcode system that allows digital multiplexed measurement of gene expression in a single panel. Moreover, it enables gene expression profiling in samples with poor RNA quality, such as formalin-fixed, paraffin-embedded (FFPE) specimens, and requires an extremely low amount of tissue (Veldman-Jones et al., 2015). Additionally, it is robust, sensitive, reproducible, easy to use, and provides rapid results in 24 h. These characteristics make this technique highly attractive for EUS sample analysis.

Our interest in focusing the analysis on tumor stromal gene biomarkers aroused from the increasing evidence that recognizes this compartment as a critical player in PDAC development, progression, and therapy resistance (Puleo et al., 2018; Neesse et al., 2019). The abundant and complex desmoplastic stroma that characterize PDAC holds a highly active cell population (which include fibroblasts, immune cells, epithelial-to-mesenchymal transition -EMT- derived cancer cells, perycites, neural cells) that exerts key pro-tumorigenic actions.

On this basis, the aims of the present study were to evaluate the adequacy of EUS-FNA acquired samples of PDAC to perform targeted transcriptome analysis by digital nCounter technology and to compare the obtained gene profile with that of surgical specimens.

## 2 Materials and methods

### 2.1 Patients

Consecutive patients with suspicion of resectable PDAC referred for EUS-FNA before surgery were prospectively included. Exclusion criteria for the study were coagulation disorders (INR >1.5, platelets <100,000), post-surgical anatomy (Roux-en-Y gastric bypass, esophagectomy, etc.) that prevented reaching the target lesion, and refusal to provide written consent. The study was approved by the Institutional Review Board of Hospital Clínic Barcelona (Nº HCB/2014/0841) and patients gave their informed consent.

### 2.2 EUS-FNA sample acquisition

EUS-FNA was performed using a linear array echoendoscope with a 19-gauge (19G) or 22G needle (EUS-3 Cook^R^ and Olympus EZ-shot^R^, respectively). Once the needle tip was introduced into the target lesion, the stylet was removed, and 5-mL suction was applied with a 10 mL syringe while the needle was moved back and forth 8–10 times within the lesion (fanning technique). The number of needle passes needed for diagnosis purposes was established based on rapid on-site evaluation (ROSE) of the sample by a cytotechnician or a pathologist. An additional pass was performed to acquire material for molecular analysis in the FFPE cell block. Pre-surgical diagnosis of PDAC was performed using the EUS-FNA material. Final diagnosis was based on the pathology report from the surgically resected specimen.

All procedures were performed under deep sedation controlled by an anesthesiologist. Patients were kept under observation for 4–8 h before discharge.

### 2.3 Processing of EUS-FNA samples for cytological diagnosis

Direct smears on glass slides were either air-dried and Diff-Quick stained to allow immediate verification of the adequacy and quality of the specimen or fixed in 95% ethanol for ulterior Papanicolaou stain. Material for molecular analysis was obtained by rinsing the needle with saline into a tube after sample smears were done together with the material obtained from an additional pass. Any residual clot or tissue in the hub of needles was removed carefully and kept in the same tube. Cell blocks were obtained by centrifugation of the tube, then the concentrated material was supported in HistoGel™ (American Master Tech, CA. United States) following the manufacturer’s instructions. The cell button was processed as a conventional biopsy and embedded in paraffin.

### 2.4 Pathological diagnosis and assessment of cell type composition in FNA cell blocks and surgical samples

Pre-surgical diagnosis of PDAC was obtained from pathological analysis of the cytological smears and FFPE cell blocks obtained by EUS-FNA. Final diagnosis was based on the pathological examination of the resected specimens. For the purpose of the study, the percentages of the different cell types present in cell blocks and surgical samples were assessed by a gastrointestinal pathologist (MC). The percentages of epithelial tumor cells and inflammatory cells were quantified using Hematoxylin and Eosin (H&E) stained slides since they are readily recognizable with this stain, whereas the percentage of cancer-associated fibroblasts (CAFs) was assessed using α-smooth muscle actin (α-SMA) immunostaining.

### 2.5 Immunohistochemistry

Immunohistochemical staining was performed using the standard protocol with the Ventana Benchmark instrument (Ventana, Tucson, AZ. United States). Briefly, 2 μm thick sections were performed from each FFPE cell block and its corresponding FFPE tumor block from surgical resections. After antigen retrieval with Cell Conditioning 1 (CC1, Ventana), and Tris-EDTA pH 9 as retrieval buffer, the ready-to-use monoclonal primary antibody α-SMA (clone 760-2833, Roche/Ventana, Tucson, AZ. United States) was incubated for 30 min, followed by the Ventana ultraview universal DAB Detection Kit-760-500. Sections were then counterstained with H&E. The immunohistochemical staining was evaluated by a gastrointestinal pathologist (MC) blind to any other information, using an optical microscope Olympus BX41 (Olympus Corporation, Tokyo, Japan). The immunostaining pattern was cytoplasmic. The muscular layer of normal vessels served as internal positive control. Negative controls without antibody disposal were used.

### 2.6 Sample processing for RNA extraction

Total RNA was extracted from the tumor contained in tissue blocks and paired surgical specimens using a RNeasy Mini Kit (QIAGEN; Cat No./ID: 74104), following the manufacturer’s instructions. Depending on the amount of tumor available, 5 to 10 µm-sections were performed and deparaffinized. Following extraction of total RNA and removal of genomic DNA, RNA was eluted (30 μL volume) and tested to ensure it met the optimal conditions (RNA concentration ≥12.5 ng/μL and purity 1.7–2.5 at OD 260/280 nm).

### 2.7 Gene expression analysis by NanoString nCounter

The NanoString nCounter gene expression system (NanoString Technologies; Seattle, WA) was used for gene expression profile using a custom designed NanosString nCounter CodeSet provided by IDT (Integrated DNA Technologies, BVBA, Belgium). The custom multiplex panel contained 52 relevant genes in PDAC, including genes mostly related to CAF, epithelial-mesenchymal transition (EMT) features and immune response (Supplementary Table S1). The panel also included 8 housekeeping genes. Extracted RNA (150 ng) samples were hybridized (without reverse transcription or amplification) with capture and reporter probes for the selected genes and assay controls according to the manufacturer protocol (MAN-C0021-01). After hybridization, samples were analyzed using the NanoString nCounter platform according to manufacturer’s instructions.

### 2.8 GSEA analysis

Gene set enrichment analysis (GSEA) analysis was performed with GSEA software (Subramanian et al., 2005) to identify enriched signatures between surgical and cytological samples. The gene expression datasets used were collections H (Hallmark gene set) and C2 (curated gene set: KEGG), publicly available at MsigDB (Liberzon et al., 2011). The standard parameters defined by Subramanian et al. were used in our analysis. The statistical significance of GSEA analysis was determined by 1000 permutations, the enrichment maps were created to significant (*p* < 0.05 and False Discovery Rate (FDR) < 0.25) gene sets.

### 2.9 Statistical analysis

Quantitative variables were expressed with median +standard deviation and range, whereas qualitative variables were expressed in percentages. Calculations were done with SPSS.

The results obtained by nCounter were normalized by R Studio using the NanoStringNorm package. All analyses used log2-transformed data. The normalized data were clustered by ward method and Euclidean distance. The features were used to scale and adjust the final heatmap to focus on patterns from important features. Two-tailed paired t-student test was performed to compare the expression of genes and signatures between two groups (EUS-FNA and surgical samples) by Graph Prism v5.0 and nSolver software v4.0. The heatmap and Principal Component Analysis (PCA) are represented by MetaboAnalyst 5.0. The volcano plot, the histogram and the boxplots, and the correlation plot were obtained by nSolver v4.0, Graph Prism v5.0 and R Studio v4.1, respectively.

## 3 Results

### 3.1 Patient population and samples

Eighteen treatment-naïve PDAC patients with cytological and posterior pathological confirmation were included. All of them underwent surgery with curative intention. Patients were 11 females and 7 men, with a mean age of 66.6 ± 11 years (range 42–84). Tumors were located in the head (*n* = 12; 66.7%), neck (*n* = 2; 11.1%), body (*n* = 2; 11.1%) and tail (*n* = 2: 11.1%) of the pancreas. Mean tumor size at EUS was 27.2 ± 7.5 mm (range 13–40 mm).

EUS-FNA samples were obtained through aspiration using 19G or 22G needles (both *n* = 9). Mean number of passes was 2 + 0.7.

### 3.2 NanoString analysis performance in EUS-FNA and surgical samples of PDAC

Nanostring analysis was performed successfully in all EUS-FNA samples. We profiled 52 representative genes of PDAC in samples acquired by EUS-FNA and their paired surgically resected specimens. mRNA was obtained from all samples and, notably, all of them passed the RNA quality control test for successful genomic analysis with the NanoString nCounter platform.

### 3.3 EUS-FNA samples and their matched surgical specimens show low overlapping gene stromal profile

**FIGURE 1:**
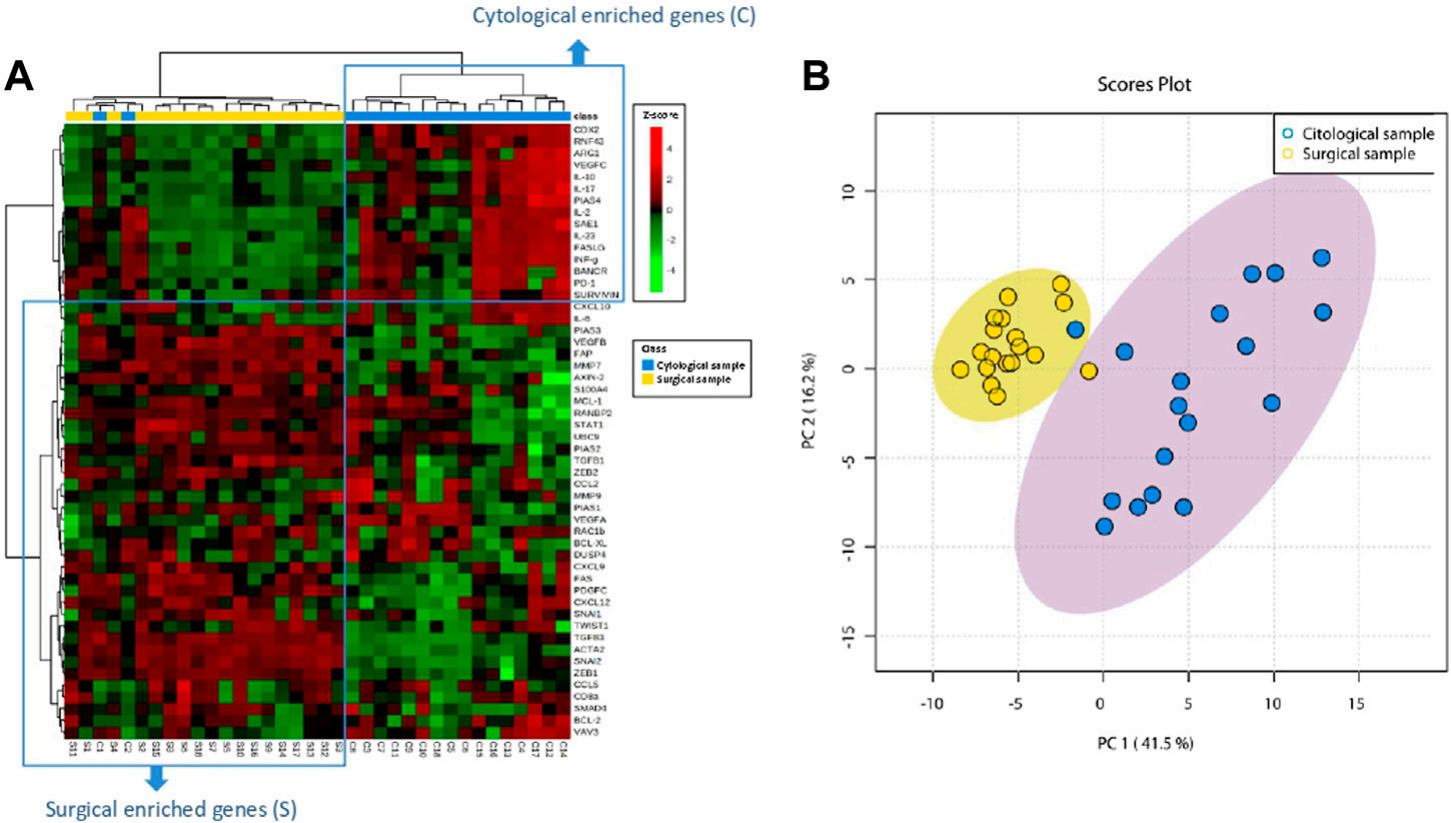
Stromal genes from PDAC are differently expressed in EUS-FNA and surgical samples. **(A)** Dendogram of hierarchical cluster analysis of relative gene expression (*y*-axis) across cytological and surgical samples. Genes and samples cluster according to their expression and similarity. On the top, the type of samples is color coded: blue for cytological samples and yellow for surgical samples. On the bottom, the type of samples as C (cytology) or S (surgical) with the corresponding patient number is shown. The color scheme represents the Z-score distribution from −4 (green, low expression) to 4 (red, high expression). Two tentative boxes (C and S) are shown. **(B)** Principal component analysis (PCA) of cytological (green area) and surgical (red area) samples according to the expression of genes (green and red circles) included in the NanoString gene panel.

Unsupervised hierarchical clustering was performed on both mRNA expression (rows) and samples (columns) and represented as a heatmap. As shown in Figure 1A, genes are distributed separately in two tentative clusters according to the type of sample (cytological or surgical). That is, cytological samples showed a cluster of over-expressed genes that showed low overlap with over-expressed genes in surgical samples. In only two patients (numbers 2 and 4) paired samples segregated together (C1 and S1, C2 and S2). The divergent transcriptomic profile observed in the hierarchical cluster analysis was also clearly appreciated when performing the principal component analysis (PCA), which separated cytological and surgical samples due to distinct gene expression (Figure 1B), revealing that they hold different stromal cell composition. Supplementary Table S2 shows the *p*-value of paired *t*-test comparing the expression of each gene between surgical and cytological samples.

### 3.4 Cytological samples obtained by EUS-FNA are enriched in immunological markers whereas surgical specimens are enriched in fibroblast and EMT-related genes

**FIGURE 2:**
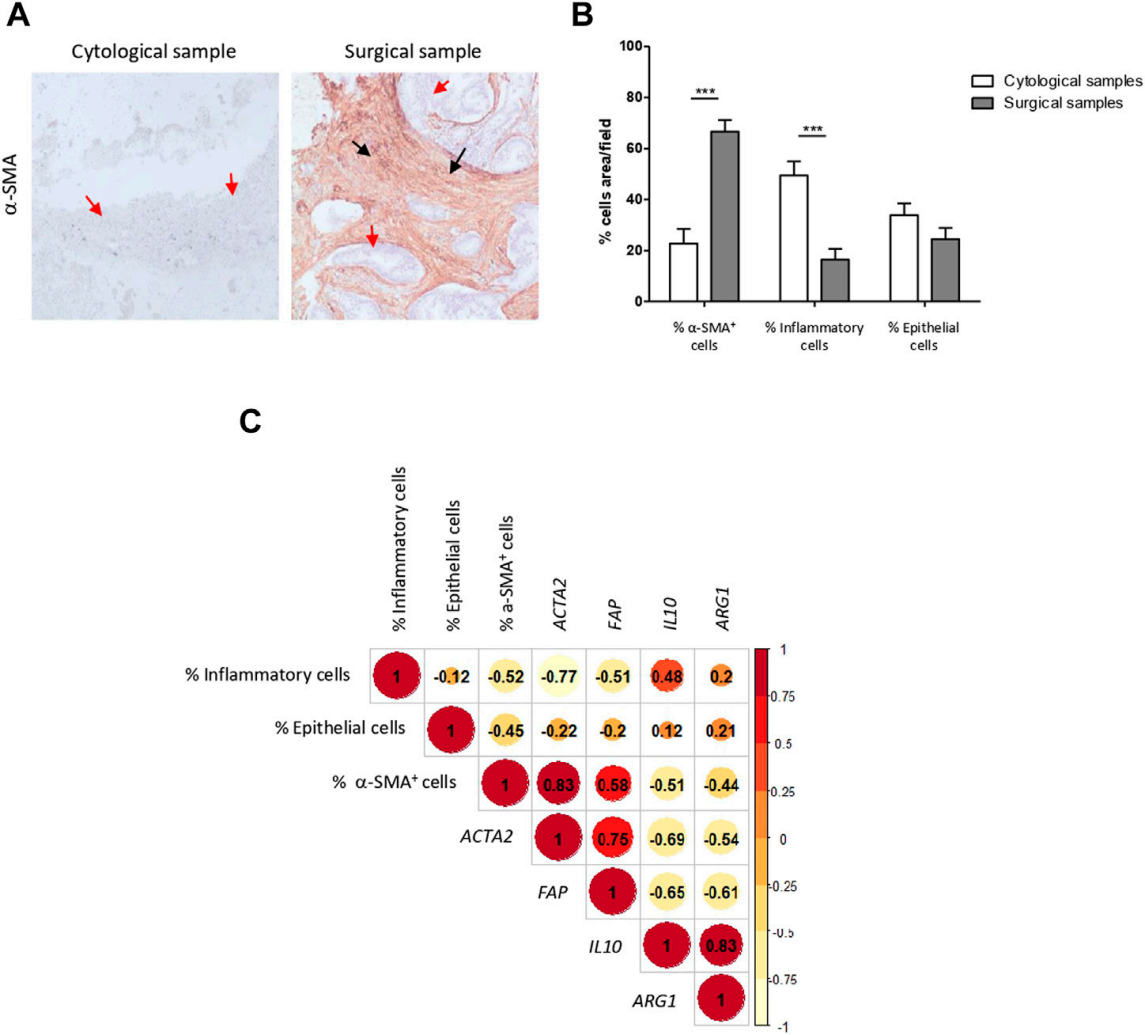
EUS-FNA samples are underrepresented in CAF and EMT related genes but enriched in immunological genes. **(A)** Volcano plot depicting gene expression differences in cytological samples *versus* surgical samples. Highly statistically significant genes fall at the top of the plot above the horizontal lines. *p*-values were adjusted for the False Discovery Rate (FDR) (adj. *p*-value). The dashed horizontal lines indicate various adj. *p*-values. The 40 most statistically significant genes are labelled in the plot. Genes were colored if the resulting adj. *p*-value was below the given *p*-value threshold based on log2 (fold-change) differential gene expression. (**B, C)** Left: Heatmap of reference genes related to CAF and EMT phenotypes **(B)** and to the immune response features **(C)** in cytological and surgical samples Right: Boxplots showing statistical differences in the relative RNA expression of selected CAF and EMT **(B)** and immune related genes **(C)** among cytological and surgical samples (asterisks indicate significant genes between cytological and surgical samples shown in the volcano plot).

Having observed the low-overlapping gene stromal profile between cytological and surgical specimens, we next looked for genes that were most differentially expressed in both types of samples. Within the 52 target genes analyzed, 30 showed statistically different expression between both types of samples (*p*-value <0.05), and 16 of them maintained their significance after False Discovery Rate (FDR) correction (adj *p*-value <0.05) (Supplementary Table S3). To identify the most differentially expressed genes, they were ranked by the log10 *p*-value of genes with different adj *p*-value and plotted against the log2 fold change in a volcano plot (Figure 2A). Among the most differentially expressed genes (adj *p*-value <0,05), those upregulated in surgical samples corresponded to genes related to CAF (such as *ACTA2, FAP, PDGFC, CXCL12*, *ZEB1*) and/or to EMT (such as *SNAI2, ZEB1, TGFB3*). Conversely, genes upregulated in cytological samples were mostly linked to the immune responses (such as *CDX2, IL10, ARG1* and *PIAS4*).

To corroborate these results, we analyzed a selection of reference genes of either CAF/EMT or immune-related features. We composed a CAF/EMT signature with well-established CAF genes expressed in human PDAC (*ACTA2, FAP, FSP, PDGFC, ZEB1, CXCL12*) (Elyada et al., 2019) and key genes related to EMT features (*SNAI2, ZEB1, ZEB2, TWIST1, TGFß1, TGFß3, STAT1*) (Gibbons and Creighton, 2018). The immune signature included genes mainly involved, although not exclusively, in immune and inflammatory processes, like chemokines (*CXCL9, CXCL10, CCL2, CCL5*), cytokines (*IL2, IL6, IL10, IL17, IL23, IFNG*), lymphocyte antigens (*CD8A*), immunosuppressive macrophages (*ARG1*) and immune checkpoints (*PD-1*). Using this approach, the segregation pattern of CAF/EMT and immune signatures remained the same. That is, genes expressed in the CAF/EMT signature remain segregated between the two types of samples, showing a clear enrichment in surgical specimens (Figure 2B). On the other hand, the immune signature was overexpressed in cytological samples, although with a less consistent expression across samples than the CAF/EMT signature (Figure 2C). In addition, GSEA analysis also suggests that surgical samples are enriched in pathways related to cancer cells and EMT, while cytological samples are enriched in immune related pathways (Supplementary Figures S1A and B). Together, these findings indicate that EUS-FNA samples from PDAC provide a low transcriptomic representation of the CAF/EMT components of the tumor.

### 3.5 Cell type composition of FNA cell blocks and surgical samples and correlation with transcriptomic data

We next sought to corroborate if the transcriptomic data obtained from bulk tissues faithfully reproduced the stromal cell composition.

**FIGURE 3:**
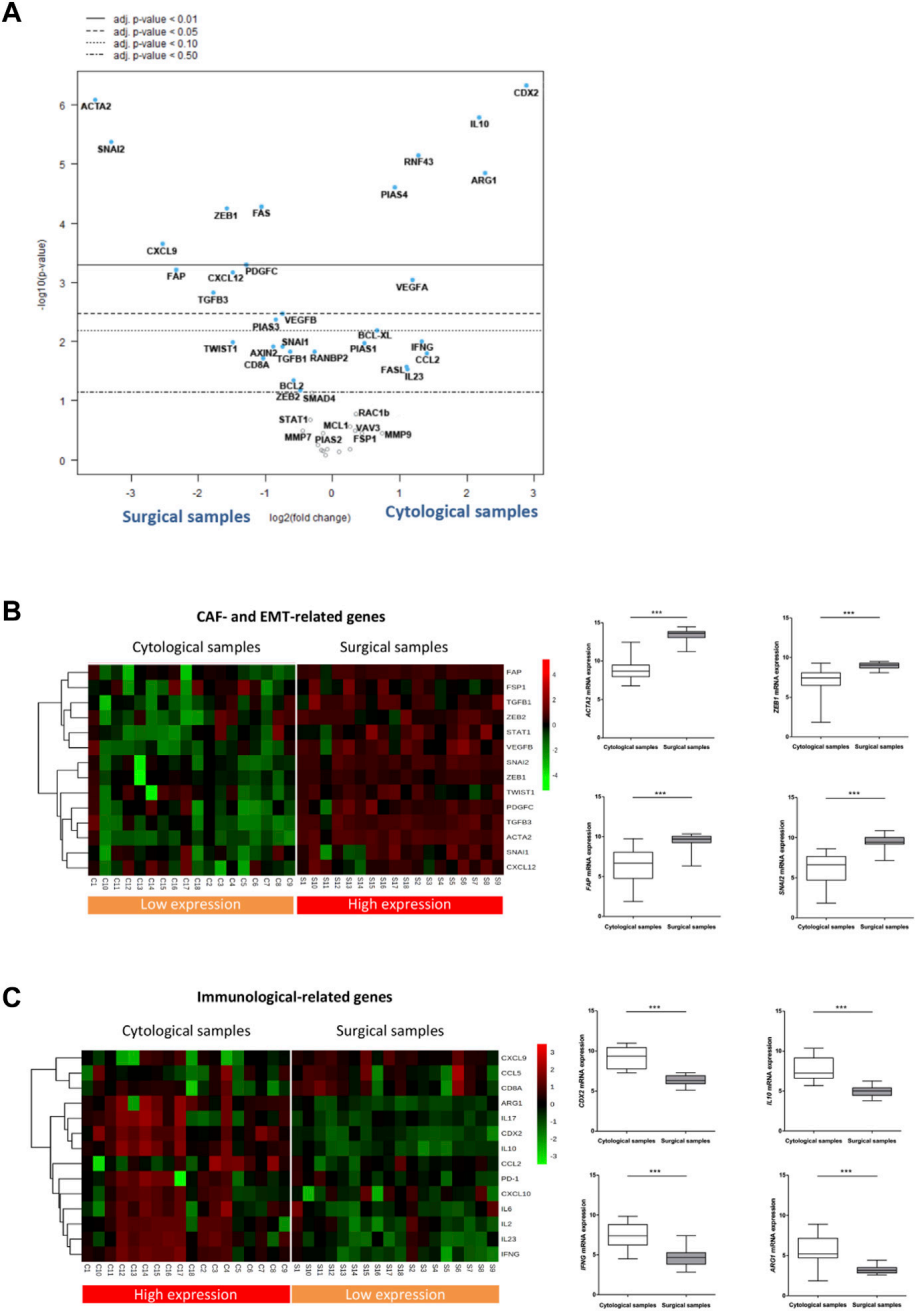
Pathological evaluation correlates with transcriptomic data confirming low stromal representation in EUS-FNA samples. **(A)** Representative images of EUS-FNA cell blocks and surgical specimens of PDAC immunostained with α-SMA. Cell block cellularity is mainly composed of epithelial cells (blue staining, red arrows), inflammatory cells on the background (green arrows), with little representation of stroma, positive for α-SMA immunostaining (brown staining, black arrows). Surgical specimens contain abundant fibroblast rich stroma with intense positivity for α-SMA (brown staining, black arrows) surrounding tumoral epithelium (blue staining red arrows). **(B)** Percentage of CAFs, immune cells and epithelial cancer cells in cytological and surgical samples (***, *p*-value <0.001). **(C)** Correlation matrix of % of stromal cell types and selected gene stromal cell markers in EUS-FNA and surgical samples. Spearman’s rank correlation method, paired.

In concordance with the transcriptomic data, H&E staining showed that EUS-FNA samples contained a scanty amount of CAFs and consisted mostly in immunological (mostly lymphocytes and plasma cells) and tumor epithelial cells. CAF abundance was also confirmed by α-SMA immunostaining. In contrast, an extensive stroma with a profuse infiltration of CAFs was a characteristic finding across tumor sections of surgical specimens (Figures 3A,B). Abundance of CAFs was confirmed by α-SMA immunostaining. Therefore, our pathological data supports the transcriptome profile differences found between cytological and surgical specimens.

We next analyzed the relationship between cell type abundance in tissue and mRNA gene expression related to pancreatic CAFs (Ogawa et al., 2021) and immune system in bulk tissue. A positive correlation was evident between the mRNA expression of *ACTA2* (gene encoding the protein α-SMA) (r = 0.83) and *FAP* (gene encoding for fibroblast activating protein, which is highly expressed in CAFs from PDAC) (r = 0.58) and the percentage of α-SMA positive CAFs. A milder correlation, although also positive, was noted between the mRNA expression of *IL10* (r = 0.48) and *ARG1* (r = 0.2) (representative immune related genes) and the percentage of immune cells (Figure 3C). Overall, both transcriptomic and pathological data confirmed that PDAC samples acquired by EUS-FNA do not provide a reliable representation of tumor stromal CAFs and EMT features, yet they afford immune biomarkers.

## 4 Discussion

In the current study we report that NanoString nCounter technology enables excellent performance of RNA transcriptomic analysis in PDAC samples obtained by EUS-FNA. Moreover, by applying a targeted NanoString panel for profiling reference tumor stromal genes, we report that EUS-FNA PDAC samples provide a low representation of CAF and EMT biomarkers, whereas they render a better map of the immunological component.

Among different levels of omic datasets, transcriptomic data has been successfully applied to respond to clinical questions for prognosis stratification and therapeutic decisions (Torres and Grippo, 2018; Turanli et al., 2021). In particular, transcriptome subtyping of PDAC has shown to better predict overall survival than standard pathological staging in patients undergoing pancreatectomy (Dreyer and Bisset, 2021). Indeed, an activated stromal subtype with a worse prognosis than the normal stromal subtype has been defined (Moffitt et al., 2015). Besides its prognostic relevance, pancreatic tumor could be transcriptionally interrogated to identify actionable targets for selective therapies (Hosein et al., 2020). Unfortunately, in real life scenario, most patients with PDAC do not undergo surgery and, therefore, resected specimens for molecular analysis are unavailable. To overcome this crucial limitation, PDAC samples acquired by EUS-FNA have been used for mRNA transcriptome profiling showing encouraging results (Laurell et al., 2006; Rodriguez et al., 2016; Bray et al., 2018; Gleeson et al., 2020; Lundy et al., 2021). Therefore, the main reason that instigated this work was the increasing awareness to integrate molecular bioanalysis into the standard endoscopic diagnostic workup in PDAC patients (Imaoka et al., 2021).

Among the variety of technologies to study gene expression, such as RNA sequencing (RNAseq), microarrays, qPCR and NanoString, the latter offers useful advantages over the rest for gene expression analysis in EUS-FNA samples, like it does not require high-quality RNA and it needs nanoscale amounts of RNA. Therefore, this technology is ideal for scenarios where only poor-quality RNA is available, including FFPE material. On the other hand, NanoString is more reliable than qPCR since it does not require reverse transcription, thereby reducing the likelihood of introducing technical variations. In addition, the NanoString platform allows for designing customized gene panels that are the basis of precision medicine (Veldman-Jones et al., 2015).

In our study, the 18 FFPE cell blocks coming from EUS-FNA samples, and their matched surgical specimens were all adequate for digital RNA analysis. Therefore, our study supports the feasibility of performing digital RNA profiling on FFPE cell blocks from standard-of-care EUS-FNA samples. To our knowledge, three studies have used NanoString technology to analyze PDAC samples acquired by EUS-FNA, reporting different rates of technical success (Gleeson et al., 2020; Lundy et al., 2021; Rasmussen et al., 2021).

To now, no studies have addressed the usefulness of RNA-based analysis of stromal representation on EUS-FNA samples of primary PDAC nor the comparison to their matched resected specimen. In our study, we designed a 52-gene NanoString panel to analyze the mRNA expression of biomarkers related to CAFs, EMT derived cancer cells and immune system, which are key cellular components of the PDAC stroma. We used FFPE cell blocks, which provide a heterogeneous sample with distinct cell types mixed at unknown proportions. To compare the expression of individual cell markers in bulk cytological and surgical samples, we applied unsupervised algorithms to profile gene expression. As said in the results, when clustering samples using the 52 genes included in the panel, 16 of the 18 pairs of samples separated by FNA or surgical origin, instead of by patient, indicating a different gene expression profile between cytological and surgical samples. Quite remarkably, a consistent and homogeneous low expression of CAF and EMT signatures was evident across FNA samples as compared to surgical specimens. This transcriptomic profile highlights that FNA do not capture fibroblasts embedded in the stromal mesh. Although without solid supporting evidence, it is generally accepted that cytological FNA samples are concentrated in tumor cells, as they are less cohesive, while stromal fibroblasts are underrepresented because they are hardly hooked to the matrix mesh. This idea was explored in an *ex-vivo* study that performed simulated FNA sample aspiration from 3 freshly resected pancreatic cancers using a syringe with a 21G needle (Crnogorac-Jurcevic et al., 2001). Needle aspiration rendered samples enriched in cancer cells, which comprised more than 95% of the total cellularity. This experimental evidence supports the low representation of CAF genes in our cohort of EUS-FNA samples.

Our study has some limitations. It could be questioned that the use of fine-needle biopsy (FNB) needles instead of FNA needles would have provided samples more enriched in fibroblasts. The reasons to use FNA needles were their good performance in our regular clinical practice for more than 20 years, the availability of ROSE and cytotechnicians’ preferences. In the view of the present results, it would be interesting to evaluate if the use of FNB needles would provide with a better representation of the stromal compartment. Moreover, it is worth mentioning that another panel of genes could have provided different results. In fact, a more comprehensive analysis to profile the whole transcriptome of the PDAC would be necessary to identify other genes of interest that can be selected for NanoString analysis.

There are still interesting issues left to explore, such as the evaluation of other genes and the performance of biopsy needles to obtain more enriched stromal samples. With this work we have reinforced the scarce evidence on the feasibility of transcriptome analysis in EUS-FNA samples and the great convenience of using nCounter technology in this kind of specimens.

## 5 Conclusion

We demonstrated an excellent performance of RNA-based analysis by NanoString in EUS-FNA samples. We believe that this is relevant since integrating molecular analysis in EUS-FNA samples may be applied in the standard clinical care for precision Oncology. Moreover, FNA needles showed poor representation of the tumor stroma, suggesting that FNB needles might be a better option for transcriptomic analysis of PDAC stroma.

## Data Availability

The data presented in the study are deposited in the Gene Expression Omnibus (GEO) repository. Accession number GSE225953. https://www.ncbi.nlm.nih.gov/geo/query/acc.cgi?acc=GSE225953.
