# Supplementary material for: Targeted transcriptomic analysis of pancreatic adenocarcinoma in EUS-FNA samples by NanoString technology
Source: Front Mol Biosci. 2023 May 17;10:1161893. doi: 10.3389/fmolb.2023.1161893 (PMC10230066; doi:10.3389/fmolb.2023.1161893)

Supplementary Material

TARGETED TRANSCRIPTOMIC ANALYSIS OF PANCREATIC ADENOCARCINOMA IN EUS-FNA SAMPLES BY NANOSTRING TECHNOLOGY

L. Pedrosa 1#, IK. Araujo 1,2#, M. Cuatrecasas 1,3,4, G. Soy 1,2, S. López 3, J. Maurel 1,4,5,6, Sánchez-Montes C 2, C. Montironi 3,7, T. Saurí 1,4,5, O. Sendino 1,2, FM. Pérez 3, F. Ausania1,4,8, G. Fernández-Esparrach 1,2,4,6, FM. Espósito 1,5, EC. Vaquero 1,6,9 † and A. Ginès1,2,4,6†*

1 Institut d’Investigacions Biomèdiques August Pi i Sunyer (IDIBAPS), Barcelona, Spain

2 Endoscopy Unit, Gastroenterology Department, ICMDM, Hospital Clínic, Barcelona, Spain

3 Pathology Department, Centre of Biomedical Diagnosis (CDB), Hospital Clínic, Barcelona, Spain

4 Facultat de Medicina i Ciències de la Salut, University of Barcelona (UB), Barcelona, Spain

5 Medical Oncology Department, Trasnlationatl Genomics and Targeted Therapies in Solid Tumors, ICMHO, Hospital Clínic, Barcelona, Spain

6 Centro de Investigación Biomédica en Red en Enfermedades Hepáticas y Digestivas (CIBEREHD

7 Molecular Biology Core, CDB, Hospital Clínic, Barcelona, Spain

8 Department of General and Digestive Surgery, ICMDM, Hospital Clínic, Barcelona, Spain

9 Gastroenterology Department, ICMDM, Hospital Clínic, Barcelona, Spain

# LP and IA have contributed equally and share first authorship.

† AG and EV have contributed equally and share senior authorship.

*** Correspondence:** Corresponding Author: Àngels Ginès. magines@clinic.cat

# Supplementary Figures and Tables

## Supplementary Tables

**Supplementary Table S1**

Gene name of 52 selected genes of the custom NanoString used.

| **NanoString-based 52 gene panel** | | |
| --- | --- | --- |
| *ACTA2* | *FSP1* | *RANBP2* |
| *ARG1* | *IL10* | *RNF43* |
| *AXIN2* | *IL17* | *SAE1* |
| *BANCR* | *IL2* | *SMAD4* |
| *BCL2* | *IL23* | *SNAI1* |
| *BCLXL* | *IL6* | *SNAI2* |
| *CCL2* | *INFG* | *STAT1* |
| *CCL5* | *MCL1* | *SURVIVIN* |
| *CD8A* | *MMP7* | *TGFB1* |
| *CDX2* | *MMP9* | *TGFB3* |
| *CXCL10* | *PDCD1* | *TWIST1* |
| *CXCL12* | *PDGFC* | *UBC9* |
| *CXCL9* | *PIAS1* | *VAV3* |
| *DUSP4* | *PIAS2* | *VEGFA* |
| *FAP* | *PIAS3* | *VEGFB* |
| *FAS* | *PIAS4* | *VEGFC* |
| *FASL* | *RAC1b* | *ZEB1* |
|  |  | *ZEB2* |

**Supplementary Table S2**

Genes and their p-value obtained comparing the gene expression between surgical and cytological samples by two-tailed paired t-test.

| **Gene** | **Paired t-test** |
| --- | --- |
| ACTA2 | 1.06E-09 |
| ARG1 | 8.40E-05 |
| AXIN-2 | 0.00799954 |
| BANCR | 0.02115632 |
| BCL-2 | 0.78327963 |
| BCL-XL | 0.46142118 |
| CCL2 | 0.28030317 |
| CCL5 | 0.37630629 |
| CD8a | 0.20416112 |
| CDX2 | 4.88E-08 |
| CXCL10 | 0.05062512 |
| CXCL12 | 0.05056104 |
| CXCL9 | 0.00202339 |
| DUSP4 | 0.79975091 |
| FAP | 5.58E-05 |
| FAS | 0.0114228 |
| FASLG | 0.00020616 |
| S100A4 | 0.14170095 |
| IL-10 | 1.13E-06 |
| IL-17 | 0.00236024 |
| IL-2 | 0.00140744 |
| IL-23 | 2.13E-05 |
| IL-6 | 0.05430368 |
| IFNG | 2.19E-06 |
| MCL-1 | 0.07634865 |
| MMP7 | 0.00333555 |
| MMP9 | 0.72078266 |
| PD-1 | 0.04519697 |
| PDGFC | 0.00101456 |
| PIAS1 | 0.61117871 |
| PIAS2 | 0.08761686 |
| PIAS3 | 4.03E-05 |
| PIAS4 | 3.57E-05 |
| RAC1b | 0.7081691 |
| RANBP2 | 0.00140463 |
| RNF43 | 1.85E-07 |
| SAE1 | 0.01105513 |
| SMAD4 | 0.14440197 |
| SNAI1 | 0.08257265 |
| SNAI2 | 2.99E-06 |
| STAT1 | 0.00278953 |
| SURVIVIN | 0.01488283 |
| TGFB1 | 0.00213478 |
| TGFB3 | 2.01E-06 |
| TWIST1 | 0.02200016 |
| UBC9 | 0.03523841 |
| VAV3 | 0.08995394 |
| VEGFA | 0.00983344 |
| VEGFB | 5.99E-05 |
| VEGFC | 0.02552835 |
| ZEB1 | 0.00045426 |
| ZEB2 | 0.00490457 |

**Supplementary Table S3**

Table presenting the most statistically significantly differentially expressed genes with the selected covariate. 'Estimated log fold-change' estimates a gene's differential expression. For categorical covariates, a gene is estimated to have 2^(log fold change) times its expression in baseline samples, holding all other variables in the analysis constant. If the covariate is continuous, for each unit increase in the selected covariate, a gene's expression is estimated to increase by 2^(log fold change)-fold, holding all other variables in the analysis constant. The 95% confidence interval for the log fold change is also presented, along with a p-value and an adjusted p-value or FDR if requested. Method column indicates the model used to estimate differential expression. Mixed negative binomial model uses the mle function to run the Wald test. The simplified negative binomial model uses the glm.nb function . The loglinear model uses the lm function. Statistical differences of differentially expressed genes are highlighted in light grey (p value < 0.05) and dark grey (False Discovery Rate adjusted p-value <0.05)

|  | **Log2 fold change** | **std error (log2)** | **Lower confidence limit (log2)** | **Upper confidence limit (log2)** | **p-value** | **adj.p.value** |
| --- | --- | --- | --- | --- | --- | --- |
| ***CDX2*** | 2.88 | 0.409 | 2.07 | 3.68 | 4.72E-07 | 8.82E-05 |
| ***ACTA2*** | -3.54 | 0.522 | -4.56 | -2.52 | 8.04E-07 | 8.82E-05 |
| ***IL10*** | 2.18 | 0.336 | 1.52 | 2.84 | 1.61E-06 | 0.000118 |
| ***SNAI2*** | -3.3 | 0.544 | -4.37 | -2.23 | 4.23E-06 | 0.000232 |
| ***RNF43*** | 1.28 | 0.219 | 0.852 | 1.71 | 7.04E-06 | 0.000309 |
| ***ARG1*** | 2.27 | 0.41 | 1.47 | 3.08 | 1.43E-05 | 0.000522 |
| ***PIAS4*** | 0.931 | 0.175 | 0.588 | 1.28 | 2.49E-05 | 0.000782 |
| ***FAS*** | -1.06 | 0.212 | -1.48 | -0.647 | 5.17E-05 | 0.00135 |
| ***ZEB1*** | -1.58 | 0.318 | -2.2 | -0.959 | 5.55E-05 | 0.00135 |
| ***CXCL9*** | -2.53 | 0.575 | -3.66 | -1.4 | 0.000225 | 0.00494 |
| ***PDGFC*** | -1.28 | 0.313 | -1.89 | -0.662 | 5.00E-04 | 0.00998 |
| ***FAP*** | -2.33 | 0.583 | -3.47 | -1.19 | 0.00061 | 0.0109 |
| ***CXCL12*** | -1.49 | 0.376 | -2.23 | -0.756 | 0.000648 | 0.0109 |
| ***VEGFA*** | 1.19 | 0.312 | 0.582 | 1.81 | 0.000922 | 0.0145 |
| ***TGFB3*** | -1.78 | 0.49 | -2.74 | -0.817 | 0.00148 | 0.0217 |
| ***VEGFB*** | -0.749 | 0.228 | -1.2 | -0.302 | 0.0034 | 0.0467 |
| ***PIAS3*** | -0.849 | 0.266 | -1.37 | -0.328 | 0.00418 | 0.0539 |
| ***BCLXL*** | 0.661 | 0.22 | 0.23 | 1.09 | 0.00649 | 0.0792 |
| ***IFNG*** | 1.33 | 0.472 | 0.405 | 2.26 | 0.01 | 0.111 |
| ***TWIST1*** | -1.49 | 0.53 | -2.53 | -0.451 | 0.0102 | 0.111 |
| ***PIAS1*** | 0.474 | 0.17 | 0.141 | 0.807 | 0.0107 | 0.111 |
| ***SNAI1*** | -0.745 | 0.272 | -1.28 | -0.212 | 0.0119 | 0.115 |
| ***AXIN2*** | -0.886 | 0.324 | -1.52 | -0.251 | 0.012 | 0.115 |
| ***TGFB1*** | -0.637 | 0.24 | -1.11 | -0.167 | 0.0145 | 0.131 |
| ***RANBP2*** | -0.272 | 0.103 | -0.474 | -0.0702 | 0.0149 | 0.131 |
| ***CCL2*** | 1.4 | 0.534 | 0.354 | 2.44 | 0.0155 | 0.131 |
| ***CD8A*** | -1.03 | 0.41 | -1.84 | -0.231 | 0.0193 | 0.157 |
| ***FASL*** | 1.1 | 0.461 | 0.193 | 2 | 0.0265 | 0.208 |
| ***IL23*** | 1.11 | 0.475 | 0.179 | 2.04 | 0.029 | 0.22 |
| ***BCL2*** | -0.583 | 0.275 | -1.12 | -0.0452 | 0.0451 | 0.33 |
| ***ZEB2*** | -0.482 | 0.25 | -0.973 | 0.00805 | 0.0669 | 0.474 |
| ***SMAD4*** | -0.302 | 0.159 | -0.614 | 0.0098 | 0.0708 | 0.486 |
| ***RAC1b*** | 0.352 | 0.245 | -0.128 | 0.831 | 0.164 | 1 |
| ***STAT1*** | -0.334 | 0.258 | -0.84 | 0.172 | 0.209 | 1 |
| ***MCL1*** | 0.259 | 0.227 | -0.187 | 0.704 | 0.268 | 1 |
| ***VAV3*** | 0.34 | 0.337 | -0.321 | 1 | 0.324 | 1 |
| ***MMP7*** | -0.447 | 0.445 | -1.32 | 0.424 | 0.325 | 1 |
| ***FSP1*** | 0.43 | 0.45 | -0.453 | 1.31 | 0.35 | 1 |
| ***MMP9*** | 0.734 | 0.777 | -0.79 | 2.26 | 0.355 | 1 |
| ***PIAS2*** | -0.149 | 0.158 | -0.459 | 0.161 | 0.356 | 1 |
| ***DUSP4*** | 0.328 | 0.372 | -0.402 | 1.06 | 0.389 | 1 |
| ***VEGFC*** | 0.273 | 0.313 | -0.34 | 0.886 | 0.392 | 1 |
| ***CCL5*** | -0.218 | 0.362 | -0.927 | 0.491 | 0.554 | 1 |
| ***IL6*** | 0.265 | 0.601 | -0.912 | 1.44 | 0.663 | 1 |
| ***UBC9*** | -0.0823 | 0.189 | -0.452 | 0.288 | 0.667 | 1 |
| ***PD1*** | -0.172 | 0.428 | -1.01 | 0.666 | 0.692 | 1 |
| ***SAE*** | -0.139 | 0.357 | -0.839 | 0.561 | 0.7 | 1 |
| ***SURVIVIN*** | 0.101 | 0.281 | -0.45 | 0.652 | 0.723 | 1 |
| ***CXCL10*** | -0.0989 | 0.442 | -0.966 | 0.768 | 0.825 | 1 |

**Supplementary Figure S1A.** GSEA enrichment plots of surgical samples vs cytological samples with representative hallmarks signatures. Normalized enrichment score (NES) and p-value are shown for each plot.


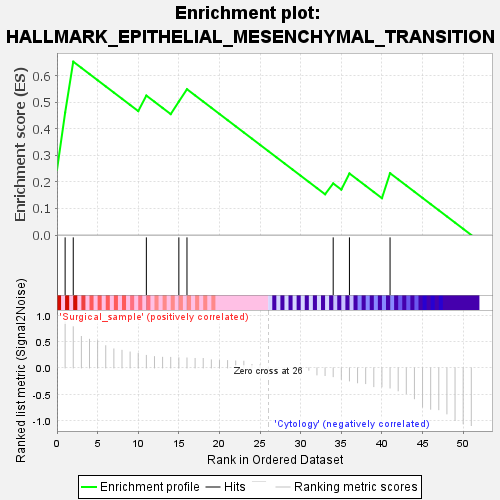


NES= 1.4630462

P-value= 0.0714


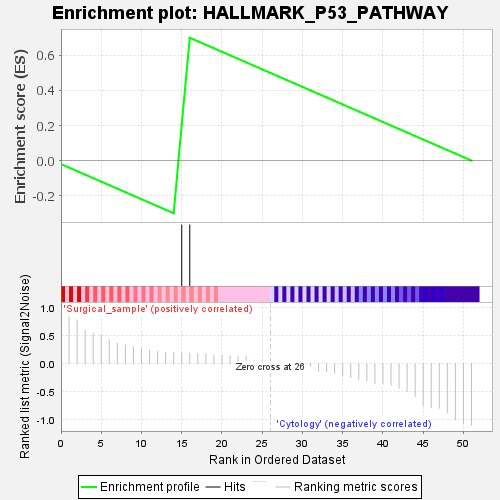


NES= 0.0222

P-value= 0.7388


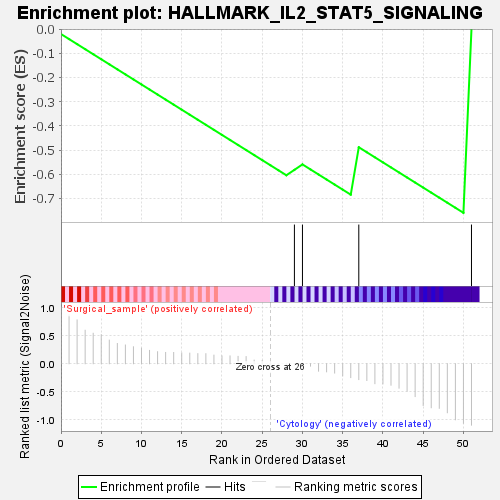


NES= -1.223

P-value= 0.222


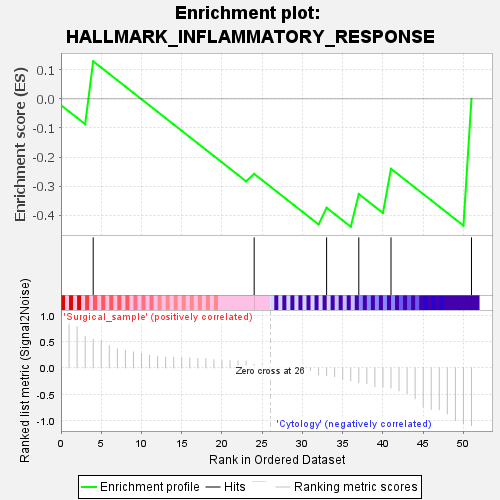


NES=-0.786

P-value= 0.702

**Supplementary Figure S1B**. GSEA enrichment plots of surgical samples vs cytological samples with representative KEGG signatures. Normalized enrichment score (NES) and p-value are shown for each plot


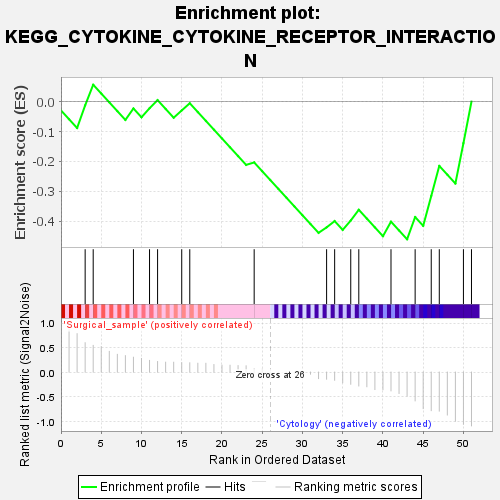

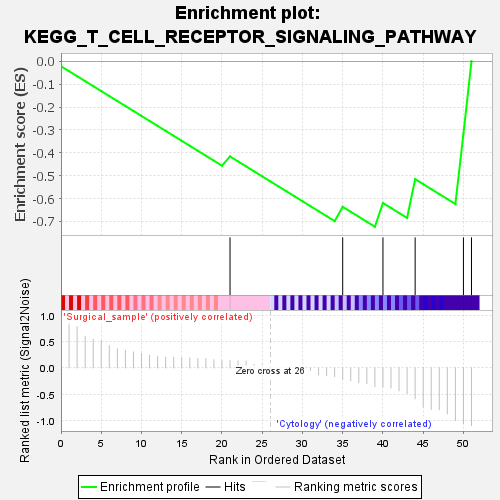

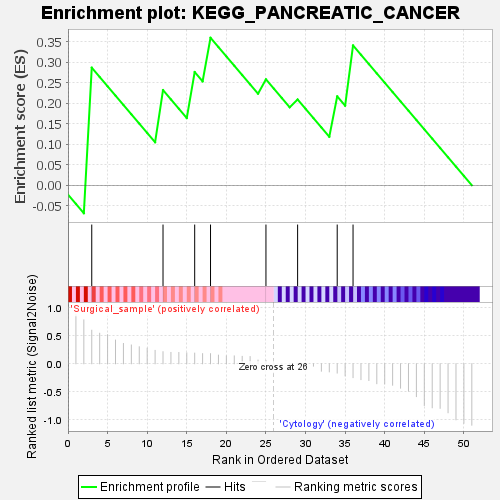

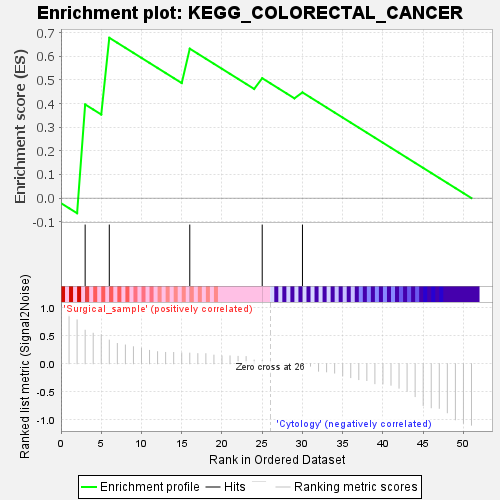

Supplement: Supplementary file 1 [file Table1.docx]
